# Determinants of Frequent Attendance in Primary Care. Study Protocol for a Systematic Review of Longitudinal Studies

**DOI:** 10.3390/ijerph17103710

**Published:** 2020-05-25

**Authors:** André Hajek, Benedikt Kretzler, Hans-Helmut König

**Affiliations:** Department of Health Economics and Health Services Research, University Medical Center Hamburg-Eppendorf, 20246 Hamburg, Germany; b.kretzler.ext@uke.de (B.K.); h.koenig@uke.de (H.-H.K.)

**Keywords:** frequent attendance, high utilization, heavy user, primary care, GP, general practitioner, systematic review, longitudinal study

## Abstract

Thus far, no study has systematically synthesized longitudinal studies investigating the determinants of frequent attendance in primary care. Consequently, the purpose of our systematic review is to give an overview of evidence based on longitudinal observational studies analyzing the determinants of frequent attendance. Three electronic databases (Medline, PsycINFO, CINAHL) will be searched. Moreover, the reference lists of studies included in our systematic review will be searched manually. Longitudinal observational studies examining the determinants of frequent attendance in primary care will be included. Disease-specific samples will be excluded. Data extraction focuses on methods (e.g., measurement of frequent attendance, statistical analysis), characteristics of the sample and key results. Furthermore, the quality of the studies included will be examined using an appropriate tool. Two reviewers will perform study selection, data extraction, and quality assessment. A meta-analysis will be conducted (if possible).

## 1. Introduction

Primary care can be defined as the “element within primary health care that focuses on health care services, including health promotion, illness and injury prevention, and the diagnosis and treatment of illness and injury” [1]. It should be noted that only a small proportion of individuals account for a large proportion of primary care visits [2]. Therefore, so-called frequent attendance (also known as heavy use or high utilization) in primary care is associated with a tremendous economic burden [3]. To date, several cross-sectional studies have identified the correlates of frequent attendance in primary care [4,5,6]. These cross-sectional studies showed, among other things, that the likelihood of frequent attendance increased with more chronic conditions and worse functioning [4,6] as well as psychological factors (such as low satisfaction with life) [5]. 

A previous systematic review [7], which focused on the determinants of frequent attendance in primary care among older adults in European studies, concluded that particular need factors such as self-rated health or chronic conditions were strongly associated with frequent attendance. More precisely, this review [7] found that severe ill health and the need for treatment were key determinants of frequent attendance in late life. Moreover, it should be noted that functional complaints (medically unexplained symptoms) are often considered as a key reason for frequent attendance in primary care [8,9].

This previous review [7] only identified one longitudinal study [10] that investigated the determinants of primary care among older adults and therefore concluded that longitudinal studies are required to identify the factors leading to frequent attendance. In recent years, more and more longitudinal studies which examined the determinants of frequent attendance have been published [11,12,13,14,15,16]. For example, based on a nationally representative longitudinal study in Germany, one of these longitudinal studies showed that the occurrence of unemployment and deteriorations in physical and mental health increased the likelihood of becoming a frequent attender [11]. Similar findings were made based on a nationally representative sample of individuals aged 40 and over in Germany [12]. More precisely, this longitudinal study found, among other things, that decreases in health-related factors were associated with an increased probability of becoming a frequent attender [12]. Using data from a representative community cohort study (Canberra region, Australia), another study showed that persistent frequent attendance was, among other things, associated with depression, disability, physical conditions and medication use [14]. Similar results were made by other longitudinal studies [15,16].

These longitudinal studies are required to gain further insights into the factors leading to frequent attendance. However, thus far, no study systematically synthesized longitudinal studies investigating the determinants of frequent attendance. Consequently, the purpose of our upcoming systematic review is to give an overview of evidence based on longitudinal observational studies analyzing this association. This knowledge may help to manage health care use and may assist in reducing the economic burden associated with frequent attendance.

## 2. Materials and Methods

This protocol for the systematic review was developed according to the Preferred Reporting Items for Systematic Reviews and Meta-Analysis Protocols guidelines [17]. We submitted it to the International Prospective Register of Systematic Reviews (PROSPERO, registration number: submitted to Prospero).

### 2.1. Eligibility Criteria

Inclusion and exclusion criteria are presented in the next sections. Prior to final eligibility criteria, a pretest will be performed (sample of 100 titles/abstracts will be screened). After the pretest, criteria will be refined (if needed).

#### 2.1.1. Inclusion Criteria

Inclusion criteria for our systematic review are:

- Longitudinal observational studies investigating the determinants of frequent attendance in primary care (irrespective of age category).
- Studies adequately assessing frequent attendance.
- Studies published in peer-reviewed journals (German or English language).

#### 2.1.2. Exclusion Criteria

Exclusion criteria for our systematic review are:

- Cross-sectional observational studies.
- Longitudinal studies not investigating the determinants of frequent attendance in primary care.
- Studies exclusively examining samples with a specific disorder (e.g., individuals with mental disorders).
- Study design other than observational.
- Assessment of frequent attendance not appropriate (for example, unclear period of time such as “frequent attendance in the last few years”).
- Studies published in language other than English or German, or not published in a peer-reviewed journal.

Three electronic databases (PubMed, PsycINFO, CINAHL) will be used in May or June 2020. We plan to update the search prior to submission. Predefined terms will be used. Our search strategy for PubMed is displayed in Table 1. Regarding location and time, no restrictions will be applied. The reference lists of the studies finally included in our work will be searched by the two reviewers manually.

### 2.2. Data Management

Endnote X7 (Clarivate Analytics (formerly Thomson Reuters), Philadelphia, PA, USA) will be used (data import). Stata 16.0 (StataCorp, College Station, TX, USA) will be used to perform a meta-analysis (if possible).

### 2.3. Study Selection Process

When the electronic and manual searches are finished, studies will be evaluated for inclusion/exclusion. This is a two-step process including title/abstract screening and full-text screening. This process is conducted by two reviewers (AH, BK). If required, disagreements will be resolved through discussion or by including a third party (HHK).

#### 2.3.1. Data Collection Process and Data Items

Data extraction will be performed by two reviewers (AH, BK). While one reviewer will extract key data, another reviewer will cross-check the data (if required, a third party (HHK) will be involved). The study authors will be contacted if required for clarification.

Data extraction mainly covers study design, tools, statistical analysis, characteristics of the sample, and key findings.

#### 2.3.2. Assessment of Study Quality/Risk of Bias

Thus far, no consensus on a quality assessment tool for health care use studies exists [18]. The evaluation of the study quality will be performed using a tool for health care use studies (Hohls et al. [18]/Stuhldreher et al. [19]). Previous studies [19,20] also used these tools [18,19]. In accordance with these studies, we will also use this tool [18] due to its conciseness, clarity and applicability. This tool [18] covers the main assessment criteria (arranged in six groups according to their topic) “scope” (e.g., “study objective”), “general HCU” (e.g., “HCU description”), “study design and analysis” (e.g., “statistics”), “presentation of results” (e.g., “demographics”), “discussion” (e.g., “limitations discussed”), and “general” (e.g., “conflict of interest stated”). For further details, please see Table Appendix 1 of Hohls et al. [18] and Appendix Table A1 of Stuhldreher et al. [19].

Both reviewers (AH, BK) will independently evaluate the study quality. In case of disagreement, discussions will be used to reach a consensus (or by including a third party, HHK). The assessment of the study quality will be integrated in our systematic review.

#### 2.3.3. Data Synthesis

A PRISMA flowchart will be used to illustrate the process of study selection. The results will be synthesized narratively. Results will be categorized according to the model of Andersen [21], distinguishing between predisposing characteristics such as age or sex, enabling resources like access to health care and need factors such as chronic conditions. A meta-analysis will be performed (if possible).

### 2.4. Patient and Public Involvement Statement

The present review protocol did not involve individual patients or public agencies.

### 2.5. Ethics and Dissemination

No primary data will be collected. Therefore, approval by an ethics committee is not required. Our findings are planned to be published in a peer-reviewed journal.

## 3. Discussion

The correlates of frequent attendance in primary care have mostly been investigated in cross-sectional studies. In recent years, some longitudinal studies examining the determinants of frequent attendance in primary care have been published. However, no study systematically synthesized longitudinal studies identifying the determinants of frequent attendance in primary care. Hence, our objective of this systematic review is to provide an overview of longitudinal observational studies examining the determinants of frequent attendance. Moreover, the quality of the studies will be evaluated. We expect that particular need factors such as worse self-rated health or chronic conditions contribute to the onset of frequent attendance longitudinally.

This is the first systematic review with regard to the determinants of frequent attendance in primary care explicitly focusing on longitudinal studies. Concentrating on longitudinal studies may help to identify studies with a high methodological quality. This focus on longitudinal studies may contribute to more valid conclusions about the factors leading to frequent attendance. Several steps (study selection, data extraction and quality assessment) will be performed by two reviewers. However, we cannot rule out the possibility that a meta-analysis cannot be performed due to heterogeneity between the studies.

## 4. Conclusions

Potential gaps in research could be identified by our systematic review. For example, it may be the case that existing longitudinal studies mainly focused on temporary frequent attenders. However, knowledge about the determinants of persistent frequent attendance may be particularly important, which for example could be examined in future studies. This may help to develop interventions to address the challenge of frequent attendance in primary care. Ultimately, this may help to reduce the economic burden caused by persistent frequent attendance in primary care.

## Figures and Tables

**Table 1:** Search strategy (PubMed).

Number	Term Entered
#1	Frequent
#2	Use*
#3	Consult*
#4	Attend*
#5	#1 AND (#2 OR #3 OR #4)
#6	Heavy use*
#7	High utili*
#8	#5 OR #6 OR #7
#9	Physicians, Primary Care
#10	Physicians, Family
#11	Family Practice
#12	General Practitioners
#13	GP
#14	#9 OR #10 OR #11 OR #12 OR #13
#15	Longitudinal
#16	#8 AND #14 AND #15
